# Influencing factors associated with the mode of birth among childbearing women in Hunan Province: a cross-sectional study in China

**DOI:** 10.1186/s12884-016-0897-9

**Published:** 2016-05-16

**Authors:** Yuhui Shi, Ying Jiang, Qingqi Zeng, Yanfei Yuan, Hui Yin, Chun Chang, Ruyan Pang

**Affiliations:** Department of Social Medicine and Health Education, School of Public Health, Peking University, Beijing, 100191 China; Chinese Center for Disease control and Prevention, Beijing, 102206 China; Maternal and Child Health Care of China Association, Beijing, 100080 China

**Keywords:** Vaginal birth, Caesarean section, Mode of birth, Health promotion, China

## Abstract

**Background:**

An unnecessary Caesarean section (CS) can cause increased maternal and perinatal morbidity and other adverse short- and long-term outcomes. However, countries worldwide have witnessed an increasing trend toward the use of CS. Our objectives were to explore the influencing factors associated with the mode of birth among childbearing women in Hunan Province and to provide evidence and suggestions for the improvement and further understanding of vaginal birth (VB) in China.

**Methods:**

A total of 977 childbearing women (375 pregnant women and 602 mothers of infants) were enrolled in this study using a two-stage cluster sampling method, and a self-administered questionnaire was used to collect data relating to the mode of birth. A *t*-test and *χ*^2^-test were used to analyse the differences between groups, and logistic regression analysis was used to explore the factors that influenced the mode of birth.

**Results:**

The VB ratio was 46.2 %, while the CS ratio was 53.8 % in Hunan Province. Among women whose preference was VB, only 69.4 % gave birth by VB. Among women whose preference was CS, 98.1 % gave birth by CS. The top four reasons for preferring CS were a lack of confidence in VB (37.3 %), an abnormality in the prenatal examination (36.6 %), the notion that the baby would suffer fewer risks (34.8 %) and the fear of pain from VB (32.7 %). Age, prenatal examination, and doctors’ suggestion were significantly associated with women’s mode of birth preference, while place of household registration, husband’s preference, prenatal examination and doctors’ suggestion had a significant influence on women who changed their choice from VB to CS.

**Conclusions:**

The percentage of CS in Hunan was extremely high. Medical factors, such as abnormalities in prenatal examinations, and non-medical factors, such as a lack of confidence in VB, the fear of pain during VB, the desire to select the time of birth and healthy birth systems, should be seriously considered. Targeted health promotion interventions should be implemented to improve the performance of VB.

**Electronic supplementary material:**

The online version of this article (doi:10.1186/s12884-016-0897-9) contains supplementary material, which is available to authorized users.

## Background

A Caesarean section (CS) can save lives and prevent injuries in circumstances such as dystocia, malpresentation and foetal distress [1, 2], which occur in approximately 5–15 % of pregnancies [3]. An unnecessary CS can cause additional maternal and perinatal morbidity in cases such as postpartum haemorrhaging, reduced fertility and placental complications in subsequent pregnancies for mothers. Additionally, there is an increased risk of postpartum respiratory morbidity, inflammatory bowel disease, obesity and Type-1 diabetes for children [4–8]. Finally, excessive unnecessary CSs are a substantial economic burden for society [9].

In the past, CS was only implemented in cases of extenuating circumstances or because of identified foetal complications. However, countries worldwide have witnessed an increasing trend toward the use of CS [10–12]. In rural China, the CS rate increased from 6 to 26 % between 1998 and 2007 [13], while in urban areas, it increased from 18.2 % in 1990-92 to 39.5 % in 1998–2002 [14]. By 2007-08, the average CS rate in China had increased to 46.2 % [15].

Studies found a disparity in the contribution of women requesting CS to the escalating CS rate in different countries, from none reported to 17 % [15–18]. It has been demonstrated that age, fear of vaginal birth (VB), issues of control, and safety of CS are the primary factors associated with women’s requests for CS [19–21]. Other researchers found that fewer interactions between the mother and practitioners [22], as well as perceived inequality and inadequacy of care [18], were also reasons that childbearing women requested CS. However, few studies exist that focus on how social and organizational factors, such as culture, hospital and family, influence women’s mode of birth preference, and a limited number of studies has focused on the change of their preference throughout the pregnancy.

Our objectives aimed to explore the influencing factors associated with mode of birth, especially women’s preference and willingness before childbirth and their actual decision, among childbearing women in Hunan Province and to provide evidence and suggestions for improvement and further understanding of VB in China.

## Methods

### Study population and recruitment procedures

A cross-sectional survey was conducted from August to September in 2012 in Hunan Province. A two-stage cluster sampling method was used to enrol the participants. Hospitals were divided into provincial, city and county levels, and two hospitals were randomly selected from each level. The following equation was used to calculate the sample size in each cluster: *n* = deff*μ_a_^2^π(1-π)/δ^2^ [23], where μ_a_ was given a value of 1.96, δ was given a value of 0.1, and deff was given a value of 2. According to a survey conducted by the health department of Hunan Province in 2011, the average CS rate in Hunan was 53.20 % [24]; thus, π was given a value of 0.532. Considering a loss rate of 5 %, the expected sample size was 202 in each cluster.

Because the mode of birth preference changes at different stages, including pregnancy and after childbirth, we selected pregnant women and mothers of infants as study subjects. Eligible subjects were defined as follows: 1) mothers of infants who were 20–45 years old and had given birth (by VB or CS) in the past year, and 2) pregnant women who were 20–45 years old and were more than 37 weeks pregnant. Those diagnosed with pregnancy-related complications, such as pregnancy-induced hypertension, gestational diabetes, etc., were excluded from our research.

Mothers of infants were investigated at postpartum clinics where they received a health examination 42 days after birth (National Basic Public Health Service Specification 2011) and at paediatric clinics in project hospitals where new mothers brought their babies for a health examination. Pregnant women were investigated at antenatal clinics where they received prenatal examinations and pregnancy classes in project hospitals.

To analyse the influencing factors associated with changes the mode of birth, the final total sample size in each cluster was 303, of which 101 were pregnant women and 202 were mothers of infants. Finally, 984 childbearing women were surveyed, including 604 mothers of infants and 380 pregnant women.

### Measures

A quantitative survey was used to conduct this research. The questionnaire content included the following (The full list of questions was provided in Additional file 1):

- Demographic characteristics: age, sex, monthly income, place of household registration, educational level, and medical insurance.
- History of pregnancy and self-reported health status. The women in the study were asked, “How many children have you given birth to?” and “How many times have you had an abortion?” Mothers of infant children who had only given birth to one child were considered “first childbirth”, and pregnant women who had never given birth before were considered “first childbirth”.
- Prenatal examination results. The women in the study were asked, “Was any abnormality found during a prenatal examination?” If the answer was yes, then the woman was asked to identify which type(s) of abnormality: ①macrosomia; ②polyhydramnios; ③foetal growth restriction; ④pelvis stenosis; ⑤malposition; ⑥gestational hypertension; ⑦ gestational heart disease(s); ⑧gestational diabetes; ⑨multiple births; or ⑩others.
- Women and their families expressed a mode of birth preference and their reasons for that preference. In this part, the preferred mode of birth was ascertained with the following question: “How would you like to give birth? 1) Vaginal birth; 2) Caesarean section.” The following question would ask why the chosen method was selected with several response options provided, each of which could be answered with yes or no.
- Only mothers of infants were asked, “How did your latest birth take place?” This question was used to understand the actual mode of birth.

### Data collection

The survey was conducted by investigators trained by a professor from Peking University’s School of Public Health using a structured questionnaire. After informed consent was gained from each participant, the participant was asked to complete an anonymous self-administered questionnaire. Investigators were required to check each questionnaire for logistical errors and blanks, and any errors and blanks were corrected on-site. Seven questionnaires were discarded due to substantial amounts of missing data. The validity rate of the questionnaire was 99.3 %, and among 977 qualified participants, 375 were pregnant women and 602 were mothers of infants.

### Ethical statement

Before conducting the survey, written informed consent was obtained from each participant in accordance with the ethical standards of the Helsinki Declaration. The study has received approval from the Peking University Institutional Review Board, and the approval number is IRB00001052-12034.

### Statistical analysis

The data were independently entered twice using Epidata 3.1, and statistical analyses were performed using SPSS 13.0. Frequencies, percentages, means and standard deviations were used to characterise the data. A *t*-test and *χ*^2^-test were used to analyse differences between groups. Based on the univariate analysis results shown in Table 1, logistic regression was used to explore the influencing factors associated with the mode of birth preference among mothers of infants and pregnant women (logistic regression 1), as well as the consistency between preference and actual mode of birth among infant mothers (logistic regression 2). In logistic regression 1, the mode of birth preference was set as the dependent variable (VB = 0, CS = 1); age, woman’s place of household registration, history of abortion, result of prenatal examination, doctors’ suggestion, self-assessed health status and whether the mother had health insurance were set as independent variables. In logistic regression 2, because pregnant women had not yet given birth, only data from mothers of infants could be used to determine the consistency between the preferred and the actual mode of birth. Whether mothers of infants who preferred VB during pregnancy changed their mode of birth choice was set as the dependent variable (no change = 0, changed from VB to CS = 1); age, women’s place of household registration, husbands’ preferred mode of birth, result of prenatal examination, doctors’ suggestion, whether the mother had health insurance, history of abortion, and self-assessed health status were set as independent variables. Backward LR was used to select the factors, and *P* < 0.05 was considered statistically significant.

**Table 1:** Basic characteristics of participants by mode of birth preference

Variables	CS^a^ (*N* = 287) n (%)	VB^b^ (*N* = 690) n (%)	*t*/*χ* ^2^ value	*P* value
Age (years)	29.4 ± 4.7	26.7 ± 3.9	8.519^c^	< 0.001
Range	19–42	18–42		
Education level				
junior middle school and below	70 (24.6)	158 (23.0)	4.801^d^	0.187
high school/secondary school	69 (24.0)	199 (29.0)		
junior college	72 (25.3)	137 (20.0)		
college or above	74 (26.0)	192 (28.0)		
Woman’s monthly income				
0-	89 (31.4)	247 (36.9)	2.661 ^d^	0.447
1020-	128 (45.2)	280 (41.8)		
3500-	48 (17.0)	101 (15.1)		
6000-	18 (6.4)	42 (6.2)		
Husband’s monthly income				
0-	14 (4.9)	54 (8.0)	4.149 ^d^	0.246
1020-	121 (42.8)	287 (42.5)		
3500-	89 (31.4)	219 (32.4)		
6000-	59 (20.9)	116 (17.1)		
Woman’s place of household registration				
Urban	168 (58.5)	357 (51.9)	3.600 ^d^	0.058
Rural	119 (41.5)	331 (48.1)		
At least one type of medical insurance				
Yes	261 (91.3)	613 (89.1)	1.037 ^d^	0.309
No	25 (8.7)	75 (10.9)		
History of abortion				
Yes	156 (55.3)	262 (38.6)	22.530 ^d^	< 0.001
No	126 (44.7)	416 (61.4)		
First childbirth				
yes	181 (63.1)	555 (80.4)	32.905 ^d^	< 0.001
No	106 (36.9)	135 (19.6)		
Result of prenatal examination				
abnormal	126 (43.9)	137 (20.1)	57.725 ^d^	< 0.001
normal	161 (56.1)	544 (79.9)		
Self-assessed health status				
good	156 (54.9)	415 (61.0)	3.087 ^d^	0.079
not good	128 (45.1)	265 (39.0)		

Note:^a^ *VB* vaginal birth, ^b^ *CS* caesarean section

^c^ *t*- test was used to compare the differences in the two groups

^d^A *χ* ^2^-test was used to compare the percentages in the two groups

## Results

### Study population

**Fig. 1:**
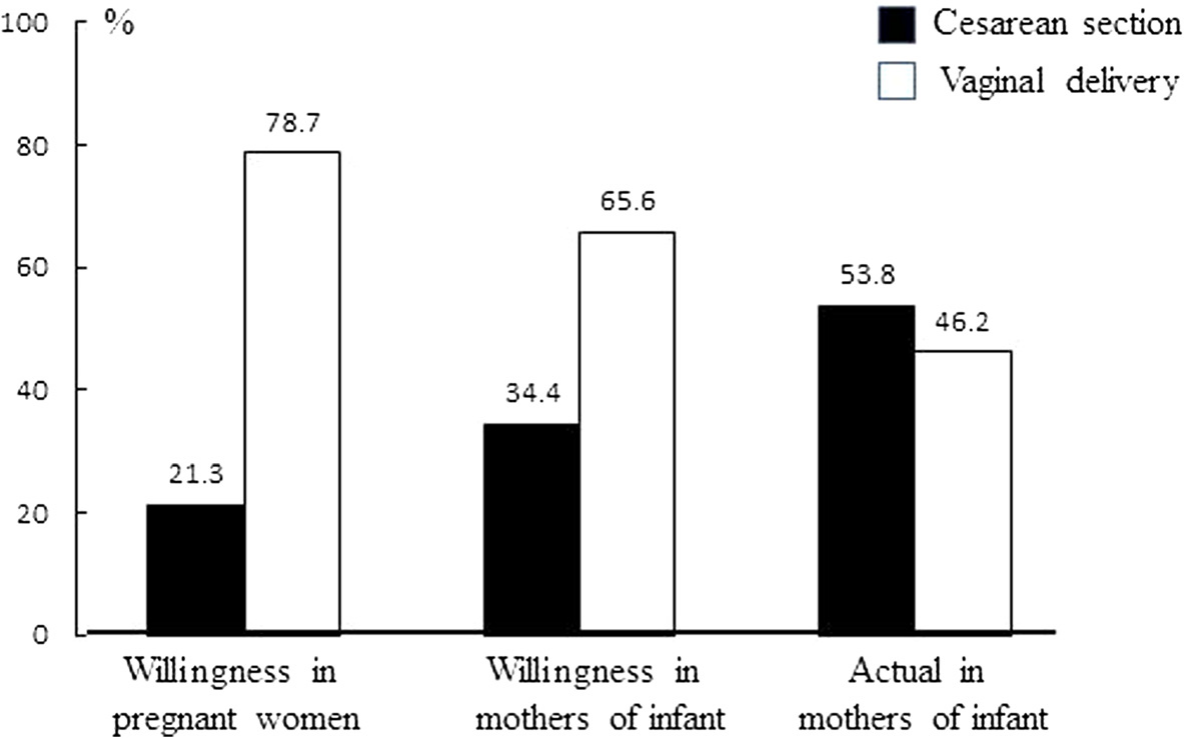
Different delivery mode choice among pregnant women and mothers of infants

Figure 1 shows that the ratio of women who preferred CS or VB changes in our study groups; 21.3 % of pregnant women preferred CS, 34.4 % of mothers of infants preferred CS, and finally 53.8 % of mothers of infants had given birth by CS, in contrast to the decreased ratio of VB (from 78.7 to 46.2 %). These results suggested that CS may not be their first choice, and other influencing factors in the pregnancy process contributed to their mode of birth change.

The average age of pregnant women and mothers of infants was (27.38 ± 4.81) years old and (27.55 ± 4.18) years old, respectively (*t* = 0.586, *P* > 0.05). Women who preferred CS were on average approximately three years older than women who preferred VB (χ^2^ = 8.519, *P* < 0.001) (Table 1). The percentage of women with a history of abortion was significantly higher among those preferring CS than among those preferring VB (χ^2^ = 22.530, *P* < 0.001). Among women preferring CS, 43.9 % experienced an abnormality in a prenatal examination, while among women preferring VB, only 20.2 % experienced an abnormality. Among women surveyed, 63.1 % of those who preferred CS and 80.3 % of those who preferred VB were first-time mothers. The economic status and self-assessed health status of women preferring CS were slightly better than were those preferring VB, but they had no statistical significance (*P* > 0.05), and the percentage of urban residents among women preferring CS was higher than those preferring VB; however, these differences were also not significant (*P* > 0.05).

### Primary reasons for mode of birth preference

Among the 676 participants who chose VB as the preferred mode of birth, the most common reason for choosing VB was that mothers would recover faster (78.7 %), and the next most common was that it was a natural process (67.2 %) (Table 2). Only 30.5 % of the participants chose VB because of its low cost. Among the 276 participants who chose CS as their preferred mode of birth, the most common reason for choosing CS was a lack of confidence in VB and concerns about VB (37.3 %), and the next most common was that an abnormality was found during a prenatal examination (36.6 %), and 10.5 % of these participants chose CS to select a fortunate time to give birth.

**Table 2:** Main reasons for the mode of birth preference among participants

Rank	Reasons	N (%)
Vaginal birth (*N* = 675)		
1	The mother will recover fast	531 (78.7)
2	Childbirth is a natural process and CS is unnecessary	454 (67.3)
3	A baby delivered by VB is healthier	403 (59.8)
4	VB is beneficial for breastfeeding	352 (52.1)
5	A baby delivered by VB is smarter	345 (51.1)
6	VB leaves no scar	266 (39.3)
7	The mother will suffer fewer risks	251 (37.2)
8	VB costs less	206 (30.5)
Caesarean section (*N* = 276)		
1	Lack of confidence and concerns about VB	103 (37.5)
2	An abnormality was found during a prenatal examination	101 (36.7)
3	The baby will suffer fewer risks	96 (34.9)
4	Fear of the pain from VB	90 (32.7)
5	Have previously had a Caesarean section	74 (26.9)
6	The mother will suffer fewer risks	59 (21.5)
7	To select the time of birth	29 (10.5)
8	Less effect on sexual behaviour	21 (7.6)

### Multivariate analysis of factors influencing mode of birth preference

The dependent variable was the mode of birth preference. Table 3 depicts the results of multivariate logistic regression analysis for the preferred mode of birth for both mothers of infants and pregnant women. Participants who found an abnormality in a prenatal examination were more likely to prefer CS (OR = 2.0, 95 % CI: 1.4–2.4), those who were advised by a doctor to have CS were five times more likely to choose VB (OR = 5.2, 95 % CI: 2.7–10.0), and older women tended to prefer CS (OR = 1.1, 95 % CI: 1.1–1.2). A woman’s abortion history and whether this was her first childbirth experience were excluded in the mode; however, further analysis showed that among women with no abortion history, 24.5 % (132/539) showed an abnormality in a prenatal examination, but among women with a history of abortion, 30.7 % (128/417) showed an abnormality (*χ*^2^ = 4.573, *P* = 0.032).

**Table 3:** Results of multivariate logistic regression of factors influencing mode of birth preference^a^

	OR	95.0 % CI for OR	
		Lower	Upper
Age	1.1	1.1	1.2
Prenatal examination (reference: normal)	2.0	1.4	2.4
Doctors’ suggestion (reference: no suggestion)			
Suggest CS	0.8	0.5	1.2
Suggest VB	5.2	2.7	10.0
No option clearly expressed	0.8	0.5	1.6
Constant	0.01		

Note: ^a^Mode of birth preference was set as the dependent variable (VB = 0, CS = 1)

Variables entered into multivariate model included age, woman’s place of household registration, history of abortion, result of prenatal examination, doctors’ suggestion, self-assessed health status and whether the mother had health insurance

### Consistency between preferred and actual mode of birth

Among the 602 mothers of infants (Table 4), 395 of them preferred VB during pregnancy, and 207 of them preferred CS. Among women whose preference was VB, 274 of them actually gave birth by VB, while 121 of them actually gave birth by CS; the consistency between the preference for VB and actual childbirth by VB was 69.4 %. Among women whose preference was CS, 203 of them actually delivered by CS, and the consistency between the preference for CS and actual childbirth by CS was 98.1 %. A significant difference was found between them (*χ*^2^ = 46.120, *P* < 0.001).

**Table 4:** Consistency between prefered and actual mode of birth (*N* = 602)

		Actual^a^		
		VB^c^	CS^d^	n
Preference^b^	VB	274	121	395
	CS	4	203	207

Note: ^a^Actual: mode of birth that mothers of infants finally selected during child birth; ^b^Preference: preferred mode of birth of mothers of infants at the last week of pregnancy; ^c^ *VB* vaginal birth; ^d^ *CS* caesarean section

### Analysis of influencing factors on the consistency between preferred and actual mode of birth

The dependent variable was whether mothers of infants who preferred VB during pregnancy changed their mode of birth choice. The results of a multivariate logistic regression analysis (Table 5) showed that the doctor’s suggestion influenced mothers to change from VB to CS (OR = 24.8, 95 % CI:3.8–161.9). The other influencing factors were the husband’s preferred mode of birth (OR = 4.4, 95 % CI:1.8–10.8) and a result of a prenatal examination (OR = 3.9, 95 % CI:2.2–6.9).

**Table 5:** Logistic regression analysis on consistency between preferred and actual mode of birth^a^

	OR	95.0 % CI for OR	
		Lower	Upper
Woman’s place of household registration (reference: urban areas)	1.7	1.0	2.9
Husband’s preferred mode of birth (reference: VB)	4.4	1.8	10.8
Prenatal examination (reference: normal)	3.9	2.2	6.9
Doctor’s suggestion (reference: no suggestion)			
Suggest VB	0.8	0.3	2.1
Suggest CS	24.8	3.8	161.9
Not clearly expressed	1.4	0.4	5.6
Constant	0.4		

Note: ^a^Whether mothers of infants who preferred VB during pregnancy changed their mode of birth choice was set as the dependent variable (no change = 0, changed from VB to CS = 1)

Variables entered into the multivariate model included age, women’s place of household registration, husbands’ preferred mode of birth, result of prenatal examination, doctor’s suggestion, whether the mother had health insurance, history of abortion and self-assessed health status

## Discussion

The CS rate in Hunan Province was much higher than the overall CS rate of 27.3 % in Asia [15], and it was also much higher than the average CS rate of 36.3 % in China [25].

Among mothers of infants who preferred childbirth by CS, 98.1 % actually gave birth by CS, while only 69.4 % of mothers of infants who preferred VB actually gave birth by VB. Another study found that women’s own preferences about CS were associated with the subsequent mode of birth [26]. Thus, strategies must be implemented to increase the preference for VB.

Many non-clinical causes related to individual social psychology characteristics played important roles in the high preference of CS. In all, 37.5 % of women who preferred CS lacked confidence and were afraid that they could not successfully give birth by VB. Existing uncertainties during the process of VB can cause intense fear of giving birth vaginally; thus, some women were willing to allow doctors to have full control of their birthing process [27, 28].

In addition to the uncertainties, fear of pain during VB was also an important factor influencing women to choose CS. To avoid the risk of emergency CS in labour after a day’s pain, they chose to undergo CS directly. Our study also found that as the time of childbirth approached, the uncertainty and fear of pain about VB increased, thus raising women’s anxiety levels, and the proportion of women who preferred CS increased. This was even more pronounced when they witnessed the great pain other pregnant women experienced during childbirth, especially those pregnant women who experienced great pain and eventually failed to give birth by VB.

Culture-related social concerns also influence the mode of birth preference. Approximately 10.5 % of women preferred CS because it allowed them to select the time of birth. In China, many people believe that a person’s fate, to some extent, is determined by the day they were born. A study found that days deemed auspicious in the Chinese lunar calendar were associated with a significantly higher probability that a CS would be performed, while those days considered inauspicious were associated with a significantly lower number of CS [29]. Age was associated with the preference of CS during pregnancy but was not associated with the change in the actual mode of birth of CS from the preferred mode of birth of VB. This was consistent with previous studies that found that older women were more likely to prefer CS [20, 30].

Multivariable analysis showed that abnormalities found in prenatal examinations (AFPE) and doctor-suggested CS were the primary influencing factors that led women to prefer CS, as well as for women who preferred VB but actually gave birth by CS. AFPE was an important medical indication for women to prefer CS and for doctors to suggest CS. Among 263 women who found an abnormality in a prenatal examination, 31.2 % were suggested CS by doctors, and 47.9 % preferred CS or agreed with their doctor to have CS. Although AFPE does not mean that CS must be chosen, doctors tend to widen CS indicators, especially when women who found an abnormality in a prenatal examination requested CS because the relationship between doctors and patients has become increasingly strained in China and because CS protects doctors from the fear of malpractice and possible litigation [31, 32].

In addition, the gap between medical costs and government investment in China is covered through paid services [33], and doctors’ incomes are directly related to the hospital revenue. Revenue-related bonus systems for doctors provide strong incentives to generate demand for profitable high-tech diagnostics, surgeries and drugs, resulting in what is known as supplier-induced demand [34, 35]. CS is obviously more profitable because of its higher cost, longer hospital stay and increased drug usage; therefore, it tends to be frequently suggested by doctors. A study showed that in China, there are 2.2 obstetric staff for every 10,000 people [36]. The serious shortage of obstetric doctors spurred doctors to prefer CS because it allowed them to schedule and reduce the birth time [37]. Policy makers should comprehensively consider the effects of supplier-induced demand on CS, including how to improve doctor-patient relationships and increase patients’ confidence in the doctors. It is especially important to reform the existing health service funding arrangements and to increase the number of obstetric doctors and midwives and their salaries to encourage them to perform vaginal births.

On the other hand, because of limited information and knowledge in the field of healthcare services, women tend to accept doctors’ advice to avoid the unknown risks. Because abortion is associated with placenta previa, placental abruption and foetal distress [38–40], it is indirectly associated with women’s mode of birth. According to Chinese statistics, the number of abortions performed in 2012 was 6.6 million [41], and a lack of knowledge and use of contraception was strongly associated with the high abortion rate [42].

### Strengths and limitations

Providing field evidence for health education and health promotion strategy development to improve natural birth in China is one of our study strengths. The data were collected from three levels of hospitals (province-city-county) so that comprehensive analysis results on the influencing factors for natural birth could be available. To reduce the bias, interviewers were trained to ensure the standardized collection of questionnaire answers, as well as to ensure standardized procedures were performed for the questionnaire survey at each hospital site.

There are some limitations of the study. First, data were collected by a questionnaire; thus, it is possible that some participants provided socially desirable responses rather than reporting their AFPE. Participants may have also over-reported their AFPE. Second, women more than 37 weeks pregnant were selected to replace the preference of mode of birth among mothers of infants in late stages of pregnancy to depict the tendency of the preferred CS rate as the pregnancy progresses. Third, the fact that only six sites were studied may limit the generalization of the findings, and the results should not be extrapolated to represent all of the pregnant women or mothers of infants in China due to important regional differences in terms of demographics and economic conditions. Fourth, there was no control group selected. Fifth, limited by resources, we only conducted a retrospective cross-sectional study, not a prospective study, to include women in earlier pregnancy stages and to follow up with them until childbirth to understand changes during the pregnancy. Finally, because of the cross-sectional nature of the data, most responses were evaluated retrospectively; therefore, recall bias was unavoidable.

## Conclusion

The CS rate in Hunan was extremely high. Influencing factors, including abnormalities in prenatal examinations, lack of confidence in VB, fear of pain during VB, the desire to select time of birth and health birth systems, should be seriously considered. Measures should be taken to cultivate awareness about the adverse short- and long-term outcomes of CS and to build confidence in VB among pregnant women. A study indicated that a standard and convenient specification for a prenatal education curriculum provided by hospitals and their doctors is appropriate for providing prenatal education to pregnant women in China [43]. Additionally, continuing professional training and development in approaches for intrapartum care among doctors and midwives should be encouraged to promote natural birth, and CS should not be considered the best method to address dystocia.

Targeted health promotion intervention activities should be implemented to improve the performance of VB and reduce unnecessary CS in China. Educational programs should be provided to women of childbearing age to cultivate awareness of the harm of abortion and to instruct them to adopt safe and effective birth control methods to reduce the abortion rate, as well as to decrease abnormalities in prenatal examinations.

Proven effective measurements should be utilized and promoted to improve women’s satisfaction and confidence with childbirth and to reduce the fear of childbirth by VB so that more women would be willing to choose or attempt VB. An effective, comprehensive program to reduce unnecessary CS should be designed and studied.

### Availability of data and materials

The dataset supporting the conclusions of this article is included within the article and its Additional files 1 and 2.

## Additional files

Additional file 1: A full List of Questionnaire. (DOC 67 kb) Additional file 2: Dataset of Survey. (XLS 1417 kb)
