# Supplementary material for: Influencing factors associated with the mode of birth among childbearing women in Hunan Province: a cross-sectional study in China
Source: BMC Pregnancy Childbirth. 2016 May 16;16:108. doi: 10.1186/s12884-016-0897-9 (PMC4869289; doi:10.1186/s12884-016-0897-9)
Supplement: Additional file 1: — A full List of Questionnaire. (DOC 67 kb) [file 12884_2016_897_MOESM1_ESM.doc]

**A questionnaire for mode of birth and its influencing factors among childbearing women in Hunan Province**

**Section 1：Socio-demographic characteristics**

1. Your age are ____ years old, and your husband’s age are ____years old.

2. Your education level is ____，and your husband’s education level is ____**（Please choose the number below）**

①Primary school or below ②Junior middle school ③High school/Secondary school ④Junior College ⑤College or above

3. Your occupation is____，and your husband’s occupation is ____**（Please choose the number below）**

①Production personnel on farming, forestry, fishery

②Production personnel on factory, transportation and equipment and relevant personnel ③Business, service personnel ④Unit in chief in government, organization, enterprise and institution ⑤ officers ⑥ Professional staff

⑦Solider ⑧ housework ⑨ Retired ⑩ Students

Unemployed Others (Please note) _______/_______

4. During the latest year, your average annual income is ____，and your husband’s income is ____**（Please choose the number below）**

①199 Yuan or below ②200-1019 Yuan ③1020-3499 Yuan

④3500-5999 Yuan ⑤6000-8499 Yuan ⑥8500 Yuan or above

5. The number of permanent family members in your home is （at least six months staying together in one year）____

6. Your place of household registration is ____**（Please choose the number below）**

① Cities in this province ② Villages in this province

③ Cities in other provinces ④Villages in other provinces

7. Which medical insurances have you joined？

①Urban employee basic medical care insurance ②Urban Residents basic medical care insurance ③New rural cooperative health system ④Commercial health insurance ⑤Maternity insurance ⑥Publicly funded free medical care ⑦None ⑧Others（Please note）_____________

**Section 2： History of pregnancy and self-reported health status**

1. What do you think about your health status?

① very bad ② bad ③ just so-so ④ good ⑤ perfect

2. Have you ever suffered from infectious diseases?

①Yes __________（Please note） ②No

3. Are you suffering from chronic diseases now?

①Yes _____________（Please note） ② No

4. How many times have you ever been pregnant ___; how many times have you ever aborted ____; how many child/children have you had__, of which how many of them belonged to preterm delivery__.

5. Which was the latest mode of birth you experienced? **(Only for pregnant woman)**

① Cesarean section ② Natural birth

**Section 3： Prenatal examination**

1. Did you do preparation well for this pregnancy? **(Only for pregnant woman)**

① Yes ② Have do preparation but not very well

③ No pregnant planning and preparations ④ Not sure

2. Have you gone to hospital for prenatal examination on time?

① Yes, every time ② Most time ③rarely ④ No prenatal examination

3. Was any abnormality found during a prenatal examination?

① Yes ②No **(skip to 6 if you choose ②)**

4. Which of the following abnormalities were found during the latest prenatal examination?

① Macrosomia ② Polyhydramnios ③ Fetal growth restriction ④ Pelvis stenosis

⑤ Malposition ⑥ Gestational hypertension ⑦ Gestational heart disease(s)

⑧ Gestational diabetes ⑨ Multiple births ⑩ Others (please note)_________

5. What suggestions did the doctor make in terms of delivery?

① No suggestions ② Suggestions for natural delivery

③ Suggestions for cesarean delivery ④ No specific suggestions

**Section 4: For pregnant woman, please select the willingness towards mode of birth and the corresponding reasons chosen by you/ your family members/ friends now. For mother of infant, Please select the willingness towards mode of birth in your last pregnant week.**

| Subjects | Ways for delivery | Reasons |
| --- | --- | --- |
| You | (1)  Natural birth | ① It is a natural process and unnecessary for cesarean section；  ② Want to experience the process of birth;  ③ The baby born by natural process is much smarter; ④ The baby born by natural process is healthier;  ⑤ The mother will suffer less risks;  ⑥ The natural birth is cheap;  ⑦ The mother will recover much fast;  ⑧ No scar on abdomen and looked beautiful;  ⑨ Beneficial for breastfeeding;  ⑩ Others___________ |
|  | (2) Cesarean section | ① Be afraid of the pain from the natural birth process;  ②Lack confidence and be worry about the natural birth process;  ③ Can choose the birth time;  ④ The baby will suffer less risks;  ⑤ The mother will suffer less risks;  ⑥Have been experienced cesarean birth; ⑦ Abnormality was found during the prenatal examination;  ⑧ Less influence to the married life;  ⑨ Easy to keep figure;  ⑩ Avoid the urinary incontinence in future;  Others___________ |
| Your husband | (1)  Natural birth | ① It is a natural process and unnecessary for cesarean birth; ②The baby born by natural process is much smarter;  ③The baby born by natural process is healthier; ④The mother will suffer less risks;  ⑤The natural birth is cheap;  ⑥The mother will recover much fast;  ⑦No scar on abdomen and looked beautiful;  ⑧Beneficial for breastfeeding;  ⑨Others___________ |
|  | (2) Cesarean section | ① Be afraid of the pain from the natural birth process;  ② Lack confidence and be worry about the natural birth process;  ③ Can choose the birth time;  ④ The baby will suffer less risks;  ⑤ The mother will suffer less risks;  ⑥ Have been experienced cesarean birth;  ⑦ Abnormality was found during the prenatal examination;  ⑧ Less influence to the married life;  ⑨ Easy to keep figure;  ⑩ Abnormality was found during the prenatal examination;  Others___________ |
| You and your husband’s parents | (1)  Natural birth | ① It is a natural process and unnecessary for cesarean birth; ②The baby born by natural process is much smarter;  ③The baby born by natural process is healthier; ④The mother will suffer less risks;  ⑤The natural birth is cheap;  ⑥The mother will recover much fast;  ⑦Beneficial for breastfeeding;  ⑧Others___________ |
|  | (2) Cesarean section | ① Be afraid of the pain from the natural birth process;  ② Lack confidence and be worry about the natural birth process;  ③ Can choose the birth time;  ④ The baby will suffer less risks;  ⑤ The mother will suffer less risks;  ⑥ Have been experienced cesarean birth;  ⑦ Abnormality was found during the prenatal examination;  ⑧ Avoid the urinary incontinence in future; ⑨ Others___________ |
| Your friends | (1)  Natural birth | ① It is a natural process and unnecessary for cesarean birth; ②The baby born by natural process is much smarter;  ③The baby born by natural process is healthier; ④The mother will suffer less risks;  ⑤The natural birth is cheap;  ⑥The mother will recover much fast;  ⑦Beneficial for breastfeeding;  ⑧Others___________ |
|  | (2) Cesarean section | ①Be afraid of the pain from the natural birth process;  ②Lack confidence and be worry about the natural birth process;  ③ Can choose the birth time;  ④ The baby will suffer less risks;  ⑤ The mother will suffer less risks;  ⑥ Have been experienced cesarean birth;  ⑦ Abnormality was found during the prenatal examination;  ⑧ The body shape is easy to recover;  ⑨ Avoid the urinary incontinence in future;  ⑩ Others___________ |

**Section 5: Mode of birth (Only for mother of infant)**

1. Which mode was the mode of birth in your latest childbirth?

① Cesarean section ② Natural birth

2. Who made the decision of the way for childbirth?

① You ② Your husband ③ Father-in-law and mother-in-law

④ Your parents ⑤The doctor ⑥ The whole family’s opinion after the discussion

⑦ Others___________

3. The date for your latest delivery was_____________, and it was the Week ____

4. Which kind of hospital did you choose for the latest childbirth?

①Province-level hospital ② City-level hospital ③ County-level hospital ④Township-level hospital ⑤Private clinic

5. Did you give trial of birth before cesarean section? (Only who selected cesarean section answer)

① Yes ② No

6. Which type of cesarean delivery did you take in the latest delivery?

① elective cesarean section ② Emergency cesarean section

7. Have you ever been experienced the following situations in your latest cesarean delivery?

① Abnormal vagina (e.g., head basin asymmetry, scar uterine) ② Production force abnormal (e.g., tried contraction of uterus) ③ Abnormal presentation (e.g.,breech presentation) ④ Abnormal premature ⑤Abnormal delivery history ⑥ Fetal factors

⑦ Pregnancy complications ⑧ Internal and surgical complications ⑨ The failure of odinopoeia or vaginal birth ⑩ Others______

8. How about your recovery after your delivery?

①Perfect ②Goof ③ Just so-so ④ Bad ⑤Terribly bad
